# Site Preferences of Copper and Cobalt Monobenzo Porphyrins in a Trans‐Dibenzo Adsorption Structure on Cu(111)

**DOI:** 10.1002/cphc.202500524

**Published:** 2025-10-28

**Authors:** Majid Shaker, Maximilian Muth, Julien Steffen, Alisson Ceccatto, Pascal Gazetas, Christoph Oleszak, Abner de Siervo, Norbert Jux, Andreas Görling, Hans‐Peter Steinrück, Ole Lytken

**Affiliations:** ^1^ Lehrstuhl für Physikalische Chemie II Friedrich‐Alexander‐Universität Erlangen‐Nürnberg Egerlandstr. 3 91058 Erlangen Germany; ^2^ Lehrstuhl für Theoretische Chemie Friedrich‐Alexander‐Universität Erlangen‐Nürnberg Egerlandstr. 3 91058 Erlangen Germany; ^3^ Lehrstuhl für Organische Chemie II Friedrich‐Alexander‐Universität Erlangen‐Nürnberg Nikolaus‐Fiebiger‐Str. 10 91058 Erlangen Germany; ^4^ Instituto de Física Gleb Wataghin Universidade Estadual de Campinas Campinas 13083‐859 SP Brazil; ^5^ Erlangen National High Performance Computing Center (NHR@FAU) Martensstrasse 1 D‐91058 Erlangen Germany

**Keywords:** Cu(111), porphyrins, scanning tunneling microscopy, statistical analysis, T‐type interaction

## Abstract

Using scanning tunneling microscopy, Cu and Co tetraphenyl monobenzo porphyrins are used as probe molecules to better understand the T‐type interactions within well‐ordered islands of Cu and Co tetraphenyl trans‐dibenzo porphyrins on Cu(111). The islands are made up of molecular rows, held together by T‐type interactions between isoindole and phenyl rings of adjacent molecules. The monobenzo molecules are found to be depleted within the bulk of the molecular rows and enriched at the edges terminating the rows. By counting over 50 000 molecules and using equilibrium considerations, the T‐type interaction energies within the trans‐dibenzo islands are estimated and the derived values are compared to values previously calculated with density functional theory, which find very good agreement for Cu‐TPtdBP but less satisfying agreement for Co‐TPtdBP.

## Graphical abstract

We find the monobenzo molecules to be depleted within the bulk of the molecular rows and enriched at the edges, terminating the rows. By counting over 50 000 molecules and using equilibrium considerations, we estimate the T‐type interaction energies within the trans‐dibenzo islands.

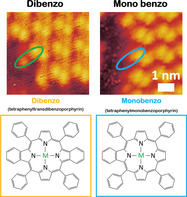

## 1 Introduction

**Figure 1:**
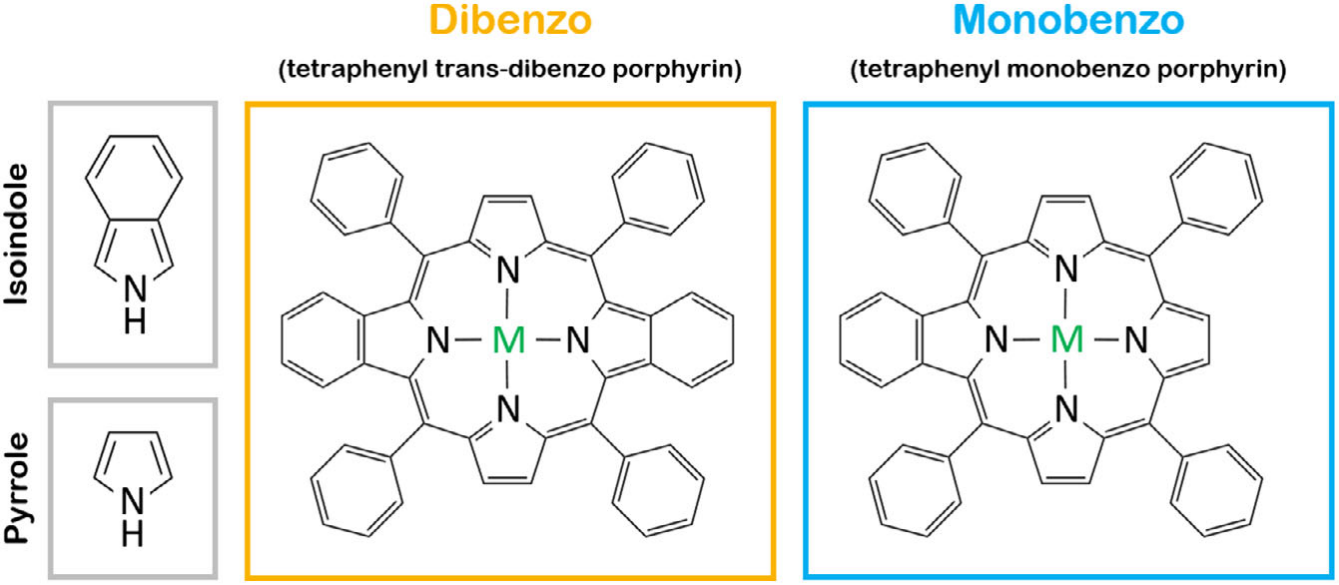
Chemical structures of tetraphenyl trans‐dibenzo porphyrin (TPtdBP) and tetraphenyl monobenzo porphyrin (TPmBP), and the isoindole‐ and pyrrole‐type functional groups, referred to throughout the text.

Porphyrins are a category of organic molecules, which can be functionalized with side groups to tailor their electronic properties and to act as structure‐forming elements.^[^ ^1^, ^2^, ^3^, ^4^ ^]^ For benzoporphyrins, benzo groups have been fused to the pyrrole groups of the porphyrin macrocycle, creating isoindole groups, see **Figure** **1**, extending the π‐system of the porphyrin molecule. This red shifts the optical spectra, increases basicity, decreases the oxidation potentials of the molecules,^[^ ^5^ ^]^ and can be used to tailor the light absorption at specific wave lengths for more‐efficient solar cells,^[^ ^6^ ^]^ or for tuning near‐infrared phosphorescence for improved imaging techniques in medicine.^[^ ^7^ ^]^

**Figure 2:**
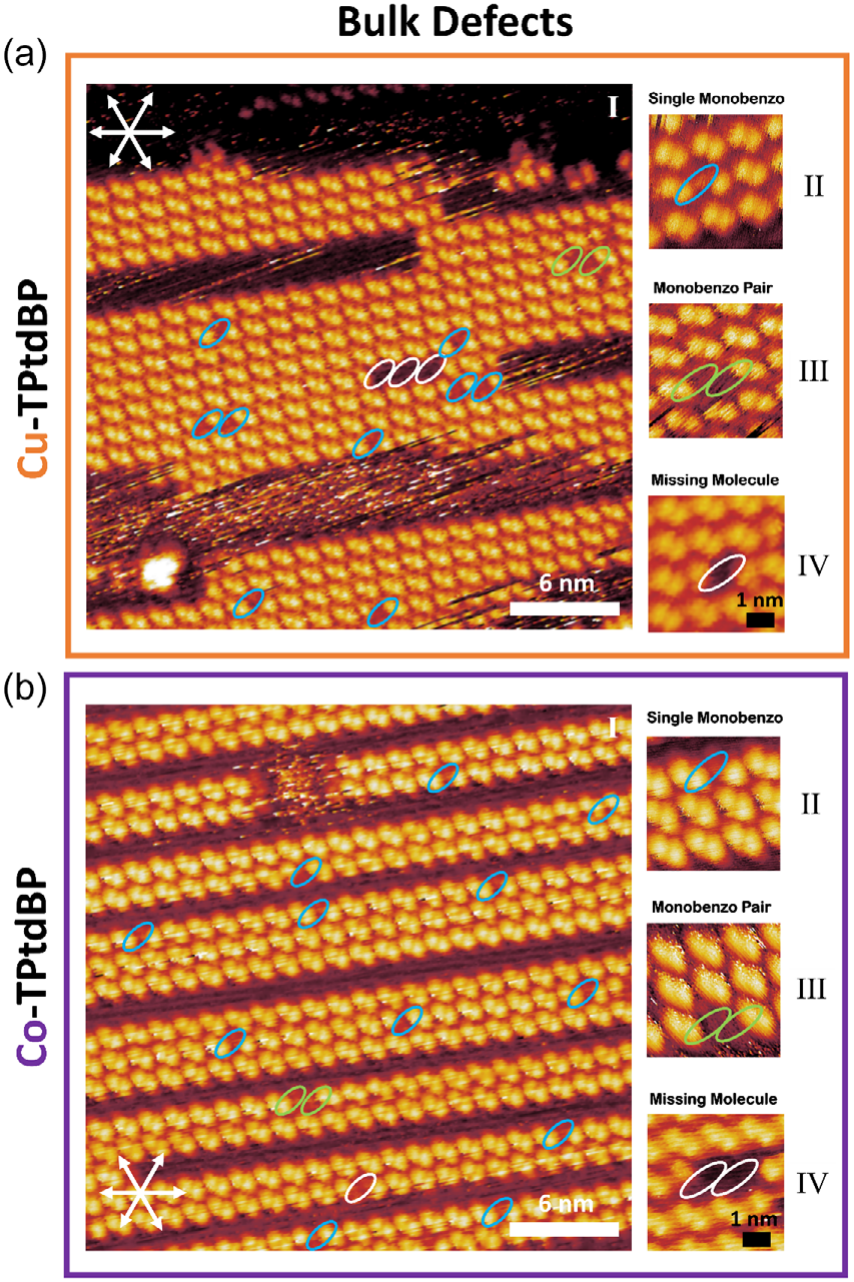
Selected STM images of a) Cu‐TPtdBP and b) Co‐TPtdBP on Cu(111) at room temperature, illustrating the defects typically observed within the molecular rows. Monobenzo molecules are visible as missing single protrusions (blue ovals; see (II)). Monobenzo pairs, with their missing isoindole groups facing each other, are visible as two missing adjacent protrusions (green ovals; see (III)). Finally, molecular vacancies are visible as two missing next‐nearest protrusions (white ovals; see (IV)). The combined concentration of defects across all measured images is tabulated in Table 1. The voltage and current values of the STM images can be found in Table S1, Supporting Information.

Because of steric repulsions between the isoindole groups and phenyl rings of copper and cobalt tetraphenyl trans‐dibenzo porphyrins (Cu‐TPtdBP and Co‐TPtdBP), the isoindole groups of the molecules are bent upwards upon adsorption on Cu(111).^[^ ^8^, ^9^, ^10^ ^]^ This allows for T‐type interactions between the upright‐standing isoindole groups and flatter‐lying phenyl rings of adjacent molecules, resulting in islands of molecular rows forming on the Cu(111) surface, rotated by 9° relative to the atomic rows of the substrate,^[^ ^8^, ^9^, ^10^ ^]^ see **Figure** **2**a (I) and b (I). Because the contrast of the molecules in the islands is given by the upright‐standing isoindole groups, Cu‐ and Co‐TPtdBP have identical appearances in scanning tunneling microscopy (STM).

T‐type interactions occur when the edge of an aromatic ring is oriented perpendicular or nearly perpendicular toward the face of another aromatic ring, slightly offset relative to the center of that ring, as known from, for instance, crystalline benzene.^[^ ^11^, ^12^ ^]^ An often observed alternative to T‐type interactions is π–π stacking,^[^ ^13^ ^]^ which is a staggered layering of the aromatic rings, where the rings are oriented parallel to each other.

Unmetalated, free‐base 2H‐TPtdBP adopts a different adsorption structure on the Cu(111) surface, with the phenyl and pyrrole rings oriented parallel to the surface.^[^ ^10^ ^]^ This places the aromatic rings of adjacent molecules in edge‐on orientations, resulting in a repulsion between adjacent molecules and no island formation on the surface, consistent with several other free‐base tetraphenyl porphyrin molecules.^[^ ^14^ ^]^

In this article, we investigate the preferred adsorption sites of small amounts of coadsorbed tetraphenyl monobenzo porphyrin within the trans‐dibenzo molecular rows on Cu(111) by counting over 50 000 molecules, using STM. Using equilibrium considerations, this allows us to extract the strengths of the T‐type interactions between the isoindole groups and phenyl rings within the molecular rows.

## 2 Results and Discussion

**Figure 3:**
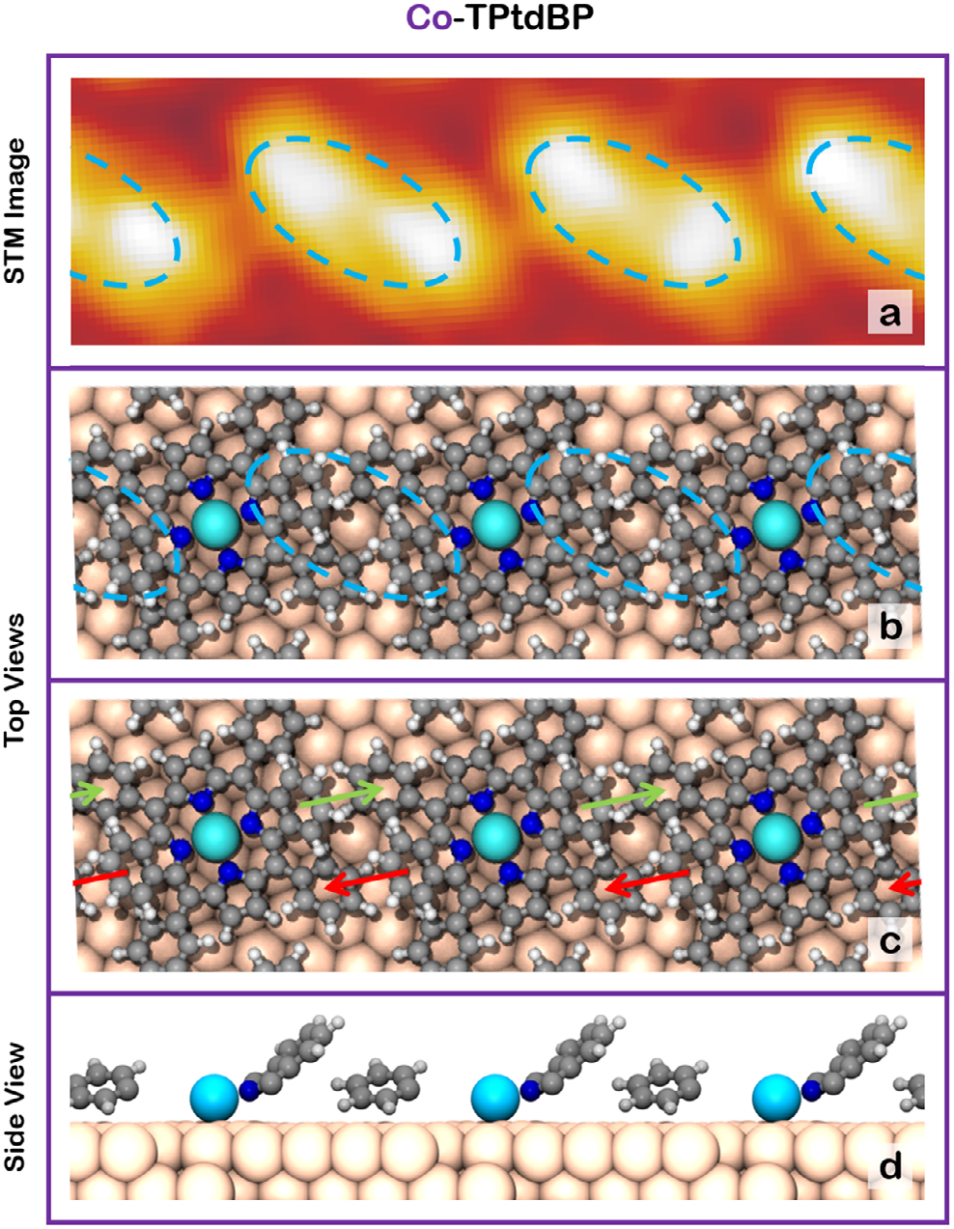
An experimentally measured a) STM image of Co‐TPtdBP on Cu(111) (–1.31 V, 51 pA), along with b) a top view of the double‐protrusion structure of Co‐TPtdBP on Cu(111) calculated by Steffen et al.^[^ ^8^ ^]^ The blue dashed ovals in (a) and (b) indicate the double protrusions resulting from isoindole groups of neighboring molecules. The red and green arrows in c) the top view of the calculated structure indicate the T‐type interactions between phenyl rings and isoindole groups of neighboring molecules. To better show the T‐type interactions within the structure, only the Co metal center and one isoindole‐phenyl‐ring pair per molecule are shown in d) the side view, and the rest of the molecule is hidden. The structure of Cu‐TPtdBP is almost identical.^[^ ^8^ ^]^ Adapted with permission from Steffen et al.^[^ ^8^ ^]^ Copyright 2025, the American Chemical Society.

Let us first focus on the internal structure of the Cu‐TPtdBP and Co‐TPtdBP islands: The islands of both molecules appear in the STM images as pairs of bright double‐protrusions in a square arrangement, forming molecular rows, see Figure 2a,b, respectively. Each protrusion is caused by an upwards‐pointing isoindole group, forming a T‐type interaction with a flatter‐lying phenyl ring of a neighboring molecule, see **Figure** **3**a–d. The T‐type interactions arrange the molecules in molecular rows oriented by 9° relative to the atomic rows of the Cu(111) substrate (see Figure 2a (I) and b (I)). Importantly, the two protrusions creating the double protrusions (blue ovals in Figure 3b) are caused by adjacent isoindole groups of different molecules. For more details on the double‐protrusion structure, we refer the reader to the work done by Shaker et al.^[^ ^10^ ^]^ and Steffen et al.^[^ ^8^ ^]^ The ordered islands are always in equilibrium with highly mobile molecules (2D gas phase),^[^ ^8^, ^9^, ^10^ ^]^ visible as streaky features, in between the ordered islands, see for instance Figure 2a (I) or b (I).

As mentioned in the Experimental Section, we know that our synthesized Cu‐TPtdBP and Co‐TPtdBP molecules contain monobenzo impurities, see Figure 1, and based on mass spectrometry data, see Figure S1, Supporting Information, we can estimate the amount of coadsorbed monobenzo molecules to be 5%–10%. This allows us to elucidate the role of the isoindole groups for the double‐protrusion structure, by identifying the preferred adsorption sites of the monobenzo molecules.

**Figure 4:**
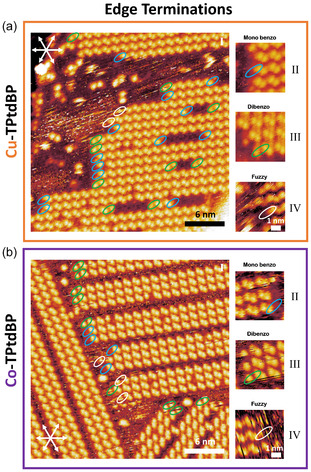
Selected STM images of a) Cu‐TPtdBP and b) Co‐TPtdBP on Cu(111) at room temperature, illustrating the terminations of the molecular rows. Monobenzo‐terminated rows are visible as rows terminating with a double‐protrusion, missing the single unpaired protrusion of the dibenzo molecules (blue ovals; see (II)). Dibenzo‐terminated rows are visible as rows terminating with a single protrusion (green ovals; see (III)). However, because of rapid attachment and detachment of molecules at the end of the rows, some rows terminate in fuzzy, stripy features, which cannot be assigned (white ovals; see (IV)). The combined numbers across all measured images are tabulated in Table 1. All voltage and current values of the STM images can be found in Table S1, Supporting Information.

Because the monobenzo molecules only contain a single isoindole group, they should be visible within the trans‐dibenzo islands as missing single protrusions. If we look at large‐scale STM images of the Cu‐TPtdBP and Co‐TPtdBP islands, see **Figure** 2, **4**, we do indeed see several missing single protrusions within the islands, consistent with the presence of a small number of coadsorbed monobenzo molecules (blue ovals in Figure 2a (II) and b (II)). We also see pairs of monobenzo molecules with their missing isoindole groups facing each other (green ovals in Figure 2a (III) and b (III)), as well as vacancies within the structure where entire molecules are missing (white ovals in Figure 2a (IV) and b (IV)). Both the monobenzo pairs and the dibenzo vacancies appear as two missing protrusions. However, whereas a monobenzo pair appears as two missing nearest protrusions, a dibenzo vacancy appears as two missing next‐nearest protrusions. The two are therefore easily distinguished.

A single monobenzo molecule within a trans‐dibenzo row is only able to form three attractive T‐type interactions, two in one direction and one in the other, compared to the four attractive T‐type interactions of a trans‐dibenzo molecule, see Figure 3c. We would, therefore, expect monobenzo molecules to be depleted within the molecular rows, compared to the nominal concentration of 5%–10% on the surface as a whole.

This is indeed the case, see **Table** **1**, which lists the observed concentrations of monobenzo molecules within the molecular rows. As can be seen, the concentrations of monobenzo molecules within the bulk of the molecular rows are 3.8% for Cu‐TPtdBP and 3.6% for Co‐TPtdBP, out of a total of more than 50 000 molecules (the details of the counting process are described in the Supporting Information, see Figure S4, Supporting Information). The two numbers are very similar, in agreement with the mass spectrometry data from Figure S1, Supporting Information, but below the nominal concentration of 5%–10% predicted by that data, confirming a depletion of monobenzo molecules within the bulk of the molecular rows.

**Table 1:** The number (concentration) of dibenzo molecules, monobenzo molecules, and monobenzo pairs facing each other, within the bulk of the double‐protrusion molecular rows, as well as the species (dibenzo, monobenzo or fuzzy) terminating the edges of the molecular rows (the details of the counting process are described in the Supporting Information, see Figure S4).

Cu‐TPtdBP				Co‐TPtdBP			
Bulk		Edges		Bulk		Edges	
Dibenzo	29 425 [96.2%]	Dibenzo	520 [31%]	Dibenzo	26 339 [96.4%]	Dibenzo	300 [53%]
Monobenzo	1 175 [3.8%]	Monobenzo	462 [27%]	Monobenzo	980 [3.6%]	Monobenzo	100 [18%]
Pairs	26 [0.08%]	Fuzzy	722 [42%]	Pairs	34 [0.12%]	Fuzzy	169 [30%]
Total	30 626	Total	1 704	Total	27 353	Total	569

However, the fact that the monobenzo molecules are only able to form a single attractive T‐type interactions in one direction should have no influence on their stability at the edges of the islands, terminating a molecular row, or in the highly mobile 2D gas phase in between the ordered islands.^[^ ^8^, ^9^, ^10^ ^]^ Compared to the bulk of the islands, the monobenzo molecules should therefore be enriched at the edges of the molecular rows and in the highly mobile 2D gas phase.

We are not able to image the concentration of monobenzo molecules in the highly mobile 2D gas phase in between the islands, but we are able to image the edges of the islands, where the molecular rows terminate. These edges are sometimes fuzzy (white ovals), indicating a rapid attachment and detachment of molecules, but frequently our resolution is good enough to distinguish between terminating monobenzo (blue ovals) and dibenzo (green ovals) molecules, as shown in Figure 4.

Table 1 lists the concentrations at the edges of the islands terminating the molecular rows. For Cu‐TPtdBP, for instance, out of 1704 terminating molecules, 31% are clearly dibenzo molecules, 27% are clearly monobenzo, and 42% are too fuzzy to tell. As expected, this shows a clear enrichment of monobenzo molecules terminating the molecular rows, compared to the 3.8%, we observe within the bulk of the molecular rows.

We can describe this as a chemical equilibrium between di‐ and monobenzo molecules within the bulk of the molecular rows and at the terminating edges of the rows.

(1) $${\text{di }}_{bulk}+{\text{mono}}_{edge}⇌{\text{di}}_{edge}+{\text{mono}}_{bulk}$$

where [di_*bulk/edge*_] and [mono_*bulk/edge*_] are the corresponding concentrations.

This assumes the bulk of the molecular rows to be in equilibrium with the terminating edges of the rows. However, because the attachment/detachment rate of molecules at the edges of the islands is much faster (timescale of a few seconds, see Video S1, Supporting Information) than the 20–40 min (1200–2400 s), we typically deposit molecules for, we expect the islands to grow in equilibrium with the highly mobile 2D gas phase around them. The STM images are, in addition, measured hours and sometimes days after deposition. This will give the islands further time to equilibrate through additional attachment/detachment at the edges of the islands as well as vacancy hopping within the islands (timescale of minutes, see Video S2, Supporting Information).

While it seems safe to assume that the vibrational entropy of di‐ and monobenzo molecules within the bulk and at the terminating edges of the molecular rows are very similar, we have to consider that the monobenzo molecule in the bulk has a twofold degeneracy (g_mono,bulk_ = 2), while the energetically favored monobenzo molecule at a terminating edge with the missing benzo group oriented away from the island has a onefold degenerate state (g_mono,edge_ = 1). This gives an equilibrium constant *K* of

(2) $$K=\frac{\left[{\text{di}}_{edge}\right]\cdot \left[{\text{mono}}_{bulk}\right]}{\left[{\text{di }}_{bulk}\right]\cdot \left[{\text{mono}}_{edge}\right]}=\frac{g_{mono,bulk}}{g_{mono,edge}}\cdot \text{exp}\left(\frac{-\Delta E}{RT}\right)$$

where ΔE is the energy difference of the exchange process, *R* is the gas constant, and *T* is the temperature (300 K).

The energy difference of the exchange process can therefore be expressed as

(3) $$\Delta E=-RT\text{ ln}\left(\frac{\left[{\text{di}}_{edge}\right]\cdot \left[{\text{mono}}_{bulk}\right]}{\left[{\text{di }}_{bulk}\right]\cdot \left[{\text{mono}}_{edge}\right]}\cdot \frac{1}{2}\right)$$

For the extreme case of all fuzzy edges being dibenzo molecules, the values from Table 1 gives an energy difference for Cu‐TPtdBP of

(4) $$\Delta E=-RT\text{ln}\left(\frac{\left(0.31+0.42\right)\cdot 0.038}{0.962\cdot 0.27}\cdot \frac{1}{2}\right)=7.3\frac{kJ}{mol}=76\;meV$$

For the other extreme, of all fuzzy edges being monobenzo molecules, the energy difference becomes

(5) $$\Delta E=-RT\text{ln}\left(\frac{0.31\cdot 0.038}{0.962\cdot \left(0.27+0.42\right)}\cdot \frac{1}{2}\right)=11.8\frac{kJ}{mol}=122\;meV$$

giving an experimentally measured energy range for Cu‐TPtdBP of 76–122 meV for the exchange process. Using the values for Co‐TPtdBP instead, also from Table 1, gives an experimentally measured energy range of 63–100 meV.

In a previous study,^[^ ^8^ ^]^ we used density functional theory (DFT) to calculate the minimum‐energy structures of both Cu‐TPtdBP and Co‐TPtdBP on the Cu(111) surface, using the experimentally observed unit cell. In addition, by freezing all atoms in the structure and removing molecules, we were able to estimate the horizontal, vertical, and diagonal interactions between molecules within the double‐protrusion structure.

This yielded a theoretically calculated strength of the attractive T‐type interactions within the Cu‐TPtdBP rows of either 170 or 210 meV per molecule, depending on the exact systems being compared, see the work done by Steffen et al. for further details.^[^ ^8^ ^]^ However, looking at Figure 3c, it becomes clear that the double‐protrusion structure has two isoindole‐phenyl interactions per molecule (marked with green and red arrows). Thus, as a dibenzo molecule within a molecular row is replaced by a monobenzo molecule, only one isoindole‐phenyl interaction is lost. The second interaction between the phenyl ring of the monobenzo molecule and the isoindole group of the neighboring dibenzo molecule remains. The theoretically predicted energy difference for Cu‐TPtdBP therefore becomes half of the calculated interaction strength per molecule, equal to 85–105 meV. This value range is extremely close to the experimentally measured range of 76–122 meV.

Considering that we neglect the differences in vibrational entropy when evaluating the experimental values and considering how the theoretically calculated values from the work done by Steffen et al.^[^ ^8^ ^]^ represent frozen molecules which are not allowed to relax after the attractive T‐type interactions have been removed, the agreement is remarkable.

For Co‐TPtdBP, however, the theoretically calculated values from the work done by Steffen et al.^[^ ^8^ ^]^ are unfortunately unreliable, giving either an attractive interaction within the rows of 150 meV per molecule or a repulsion of −70 meV, depending on the systems being compared. This is most‐likely not a limitation of the DFT calculations by Steffen et al., but rather an indication that the horizontal, vertical, and diagonal interactions within the double‐protrusion structure, are not independent for Co‐TPtdBP. Attempting to extract them as independent properties is, therefore, fundamentally flawed, and gives conflicting values.

However, assuming the attractive interaction of 150 meV per molecule to be more probable than the −70 meV repulsion, the theoretically predicted reaction energy for Co‐TPtdBP becomes 75 meV, in good agreement with the experimentally measured range of 63–100 meV.

Occasionally, we also see pairs of monobenzo molecules with their missing isoindole groups facing each other (green ovals in Figure 2a (III) and b (III)). This arrangement will result in the loss of one additional isoindole‐phenyl interaction, compared to a dibenzo neighbor. Compared to two isolated monobenzo inclusions, however, the number of isoindole‐phenyl interactions does not change. We would therefore not expect pair formation to be favored over isolated inclusions.

For a monobenzo concentration of X, we would therefore expect a concentration of monobenzo pairs $X_{pairs}$ of

(6) $$X_{pairs}=\frac{X^{2}}{2}$$

corresponding to the probability that the neighboring molecule, on the missing isoindole side, is a monobenzo molecule (*X* ^2^), facing the right direction $\left(\cdot \frac{1}{2}\right)$.

The concentration of monobenzo pairs for Cu‐TPtdBP (X = 0.038) should therefore be 0.07% and for Co‐TPtdBP (*X* = 0.036) 0.06 %, fairly close to the experimentally obtained values of 0.08% and 0.12%, see Table 1.

The enrichment of monobenzo molecules at the terminating edges of the rows can also help explain the thermal evolution of the double‐protrusion structure, observed by Muth et al.,^[^ ^9^ ^]^ as the Cu(111) surface is annealed stepwise from room temperature to 423 K. They find that the shapes of the double‐protrusion islands first become rough and irregular at 398 K before breaking completely apart at 423 K. This is most‐likely related to a ring‐fusion reaction taking place at this temperature, where the phenyl rings fuse to either the pyrrole or the isoindole rings.^[^ ^9^ ^]^ The ring fusion will flatten the molecules, especially if the ring fusion is to the isoindole groups, and thereby hinder the T‐type interaction holding the double‐protrusion structure together. A partially ring‐fused dibenzo molecule should therefore behave very similar to a monobenzo molecule and become strongly enriched at the edges of the islands terminating the molecular rows. This will thermodynamically stabilize the terminations of the molecular rows, in the same way monobenzo molecules do, and thereby stabilize the rough and irregular islands observed by Muth et al.^[^ ^9^ ^]^ with more terminated rows. As the dibenzo molecule becomes fully ring‐fused, the isoindole‐phenyl interactions are lost on both sides of the molecules, and the double‐protrusion structure breaks completely apart, as observed by Muth et al. at 423 K.^[^ ^9^ ^]^

## 3 Conclusions

As Cu and Co tetraphenyl trans‐dibenzo porphyrins are adsorbed on Cu(111) at room temperature, islands with a double‐protrusion structure are formed.^[^ ^8^, ^10^ ^]^ These islands are composed of molecular rows, held together by T‐type interactions between isoindole and phenyl rings of adjacent molecules,.

Using STM, we have determined the equilibrium concentrations of small amounts of tetraphenyl monobenzo porphyrin within the bulk of the molecular rows, and at the terminating edges of the rows of the double‐protrusion dibenzo structure.

Because the monobenzo molecules only have one isoindole group and are, therefore, missing one isoindole‐phenyl interaction compared to the dibenzo molecules, the monobenzo molecules are depleted within the bulk of the molecular rows and enriched at the edges terminating the rows.

The concentrations within the bulk of the molecular rows and at the terminating edges of the rows, therefore, allow us to estimate the strength of the isoindole‐phenyl interactions holding the double‐protrusion structure together, and compare those with previous values calculated by DFT.^[^ ^8^ ^]^ For Cu‐TPtdBP, the agreement between our experimentally predicted values of 76–122 meV and the theoretically calculated values by Steffen et al.^[^ ^8^ ^]^ of 85–105 meV is exceptionally good. For Co‐TPtdBP, the DFT calculations by Steffen et al.^[^ ^8^ ^]^ predicted either a 75 meV attraction or a –35 meV repulsion per isoindole‐phenyl interaction, indicating a problem with separating the isoindole‐phenyl interactions from all the other contributions to the adsorption energy. However, 75 meV is in good agreement with experimentally obtained values of 63–100 meV.

## 4 Experimental Section

All STM measurements were performed at room temperature, using an RHK UHV VT STM 300 with RHK SPM 1000 electronics, in an ultrahigh‐vacuum chamber with a background pressure in the low‐to‐mid 10^−10^ mbar range. We used a Pt/Ir tip in constant current mode, with the bias voltage applied to the sample. Background subtraction and mild fast fourier transformation (FFT) filtering were done with Gwyddion.^[^ ^15^ ^]^

The Cu(111) single crystal was cleaned by 600 V Ar^+^ sputtering followed by annealing to 850 K for 1–5 min, and Cu‐TPtdBP and Co‐TPtdBP, were evaporated, with the samples at room temperature, from quartz crucibles at 650–670 K. The cleanliness of the surface was verified by STM. All tunneling voltage and current values are provided in Table S1, Supporting Information.

Cu‐TPtdBP and Co‐TPtdBP were synthesized and characterized with UV–Vis, X‐ray photoelectron spectroscopy (XPS), and mass spectrometry, as described previously; see Ref. [8, 10] and the corresponding Supporting Information. From mass spectrometry characterizations of the synthesized Cu‐TPtdBP and Co‐TPtdBP molecules, see Figure S1, Supporting Information, we know that both molecules contain a small amount of monobenzo impurities. The monobenzo impurities are visible, when evaporating the molecules directly into a mass spectrometer, as a peak 50 mass units below the peak of the intact molecule, consistent with one missing benzo group. The ratio of mono‐ to dibenzo molecules desorbing from the evaporator changes drastically with time, from 35%, when freshly filled, to 5%, when almost empty, as the monobenzo molecules are slowly being distilled away, see Figure S1, Supporting Information. Based on the degassing we used before depositing molecules from a freshly filled evaporator, we estimate the amount of codeposited monobenzo molecules to be 5%–10%. The monobenzo impurities were not planned for, but gave us a unique opportunity to study the role of the isoindole groups in the double‐protrusion structure, by recording the local concentration of monobenzo molecules in different adsorption sites within the structure.

## Conflict of Interest

The authors declare no conflict of interest.

## Supporting information

Supplementary Material

## Data Availability

The data that support the findings of this study are openly available in Zenodo at https://doi.org/10.5281/zenodo.15957073.
