# Supplementary material for: Site Preferences of Copper and Cobalt Monobenzo Porphyrins in a Trans‐Dibenzo Adsorption Structure on Cu(111)
Source: Chemphyschem. 2025 Oct 28;26(22):e202500524. doi: 10.1002/cphc.202500524 (PMC12640668; doi:10.1002/cphc.202500524)
Supplement: Supplementary file 1 — Supplementary Material [file CPHC-26-e202500524-s001.zip › cphc70119-sup-0001-SuppData-S1.pdf]

SUPPORTING INFORMATION

Site preferences of copper and cobalt monobenzo porphyrins in a trans-dibenzo adsorption structure on Cu(111)

Majid Shaker,[a] Maximilian Muth,[a] Julien Steffen,[b] Alisson Ceccatto,[a]
Pascal Gazetas,[c] Christoph Oleszak,[c] Abner de Siervo,[d] Norbert Jux,[c] Andreas Görling,[b,e]
Hans-Peter Steinrück [a,*] and Ole Lytken [a,*]

*) corresponding authors: ole.lytken@fau.de, hans-peter.steinrueck@fau.de

[a] Lehrstuhl für Physikalische Chemie II, Friedrich-Alexander-Universität Erlangen-Nürnberg, Egerlandstr. 3, Erlangen 91058, Germany

[b] Lehrstuhl für Theoretische Chemie, Friedrich-Alexander-Universität Erlangen-Nürnberg, Egerlandstr. 3, Erlangen 91058, Germany

[c] Lehrstuhl für Organische Chemie II, Friedrich-Alexander-Universität Erlangen-Nürnberg, Nikolaus-Fiebiger-Str. 10, Erlangen 91058, Germany

[d] Instituto de Física Gleb Wataghin, Universidade Estadual de Campinas, Campinas 13083-859, SP, Brazil

[e] Erlangen National High Performance Computing Center (NHR@FAU), Martensstrasse 1, D-91058 Erlangen, Germany

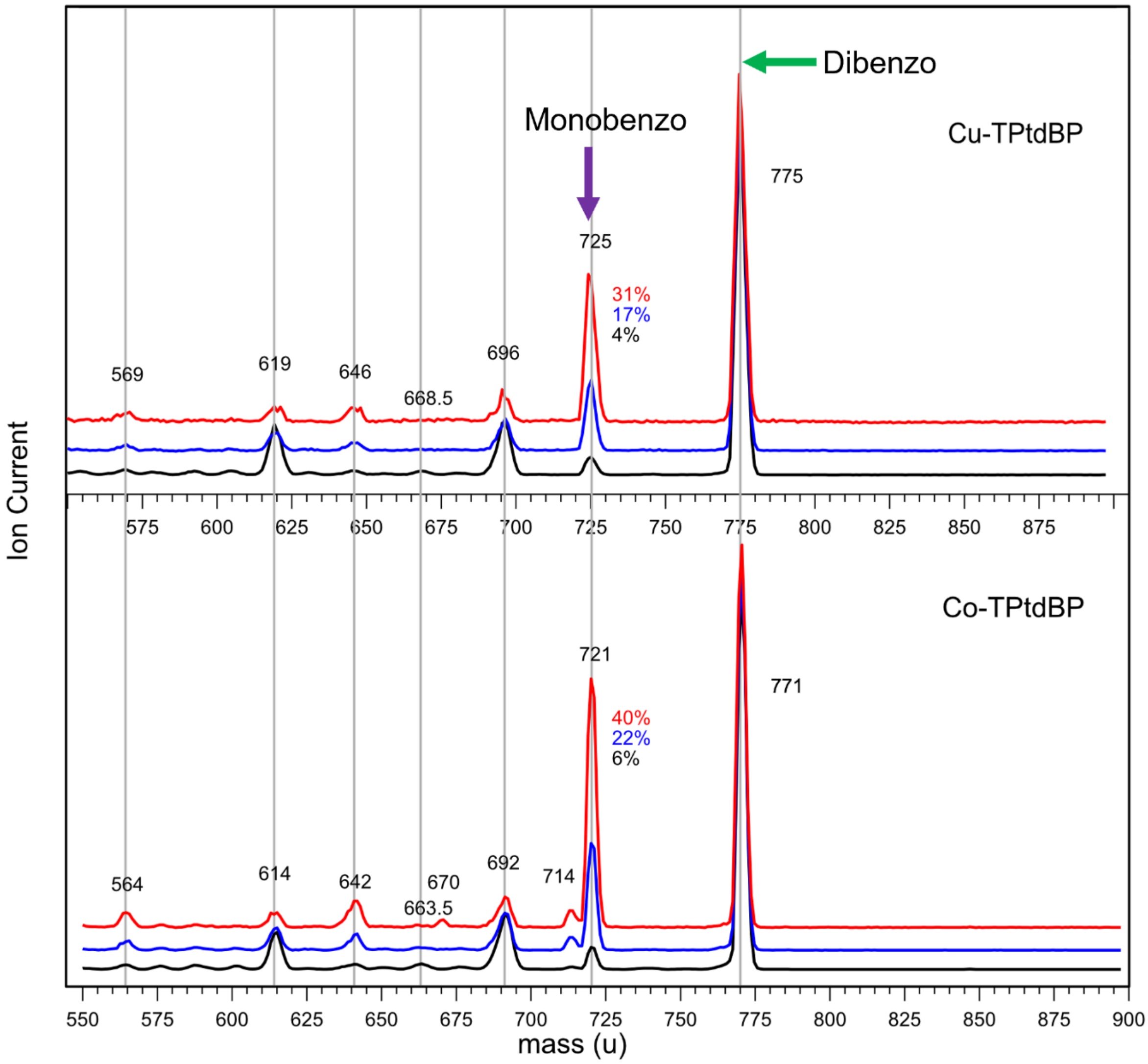

Figure S1. Normalized mass spectra of the synthesized Cu and Co TPtdBP molecules. The red spectra were measured after filling the evaporator with the molecules without degassing. The blue spectra were measured after a short degassing. The peaks at centered at 775 and 771 amu corresponds to Cu- and Co-TPtdBP, respectively, while the peaks centered at 725 and 721 amu, corresponds to the monobenzo impurities. The small peak at 714 amu is a free-base 2H-TPtdBP impurity, while the peaks at lower masses with a repetition unit of 77 – 80 amu are most-likely a cracking pattern of the main peaks, related to a loss of phenyl rings. Based on the degassing of the evaporator before depositing molecules from a freshly-filled evaporator, we estimate the amount of codeposited monobenzo molecules to be 5 – 10 %.

Table S1. Voltage and current values of the STM images shown in Figures 2 and 4.

	**Voltage (V)**	**Current (pA)**
**Figure 2**	**Bulk Defects**	
a-I	-1.02	32
a-II	-1.25	56
a-III	-1.00	44
a-IV	-1.32	71
b-I	-1.01	35
b-II	-1.01	34
b-III	-2.00	54
b-IV	1.76	42
**Figure 4**	**Edge Terminations**	
a-I	-1.32	70
a-II	-1.32	71
a-III	-1.32	70
a-IV	-1.42	49
b-I	-1.26	53
b-II	-1.31	39
b-III	-1.31	39
b-IV	-1.26	54

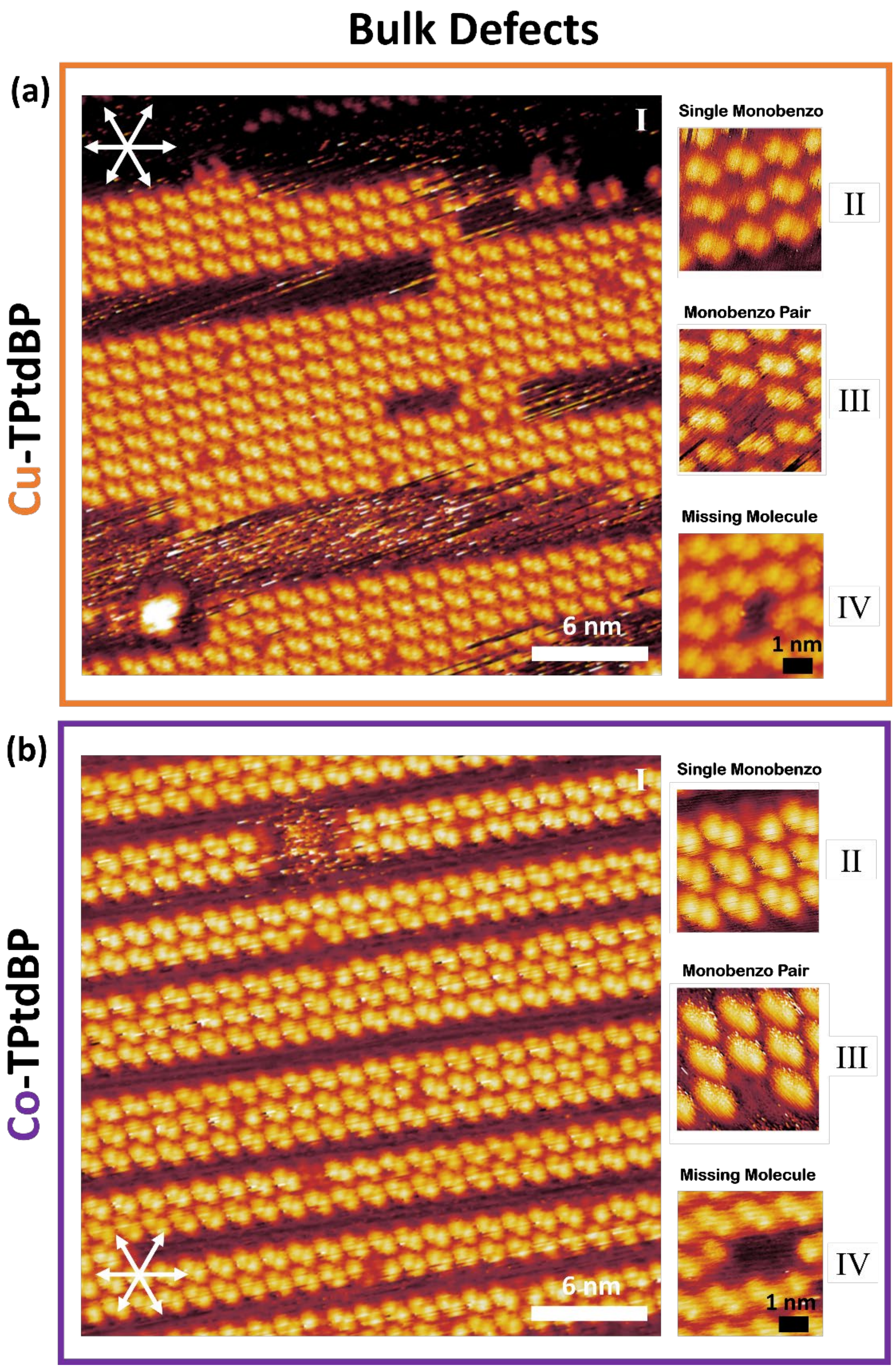

Figure S2. Figure 2 of the manuscript without ovals.

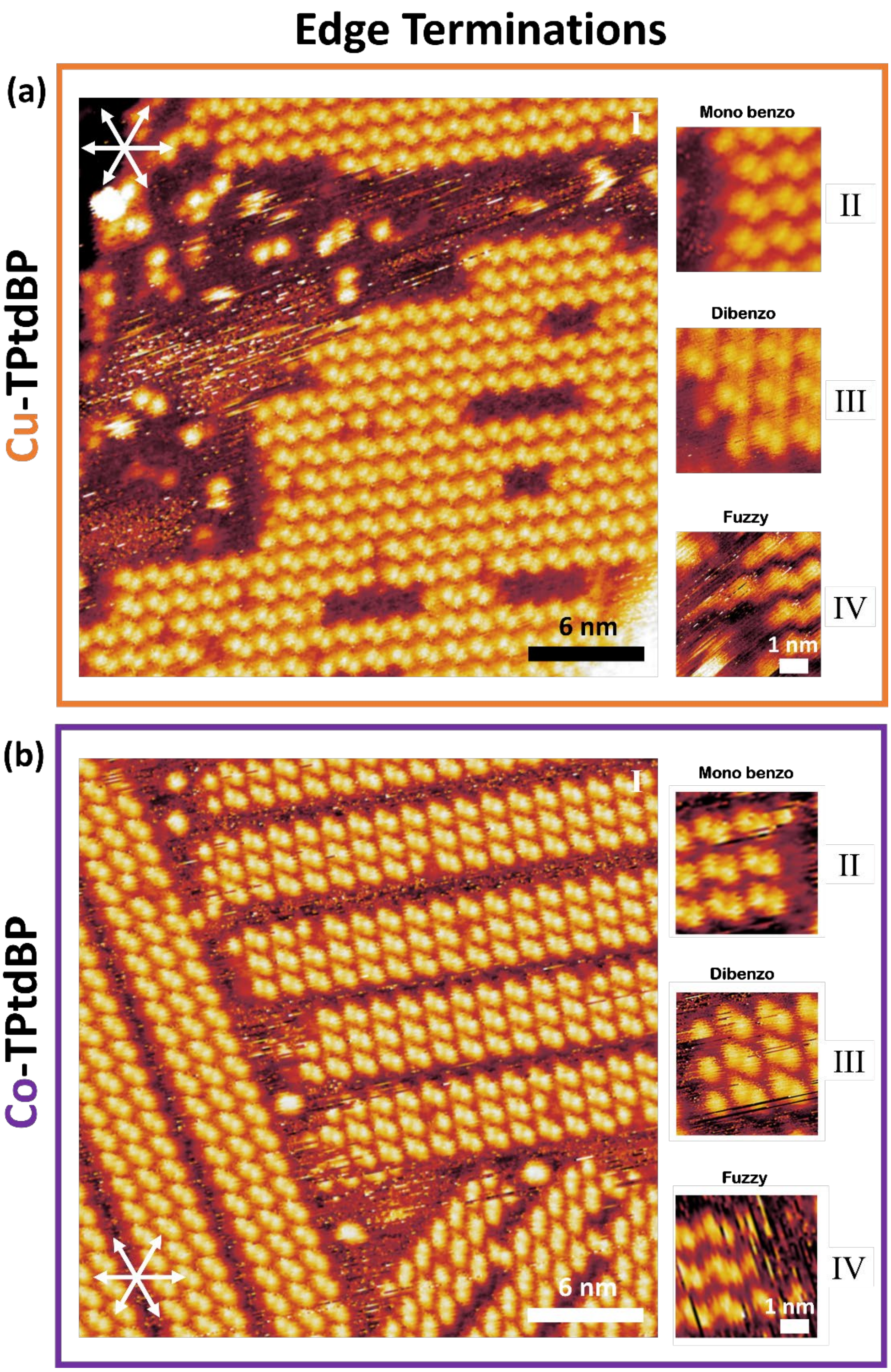

Figure S3. Figure 4 of the manuscript without ovals.

Counting of molecules: We analyzed a total of 78 STM images of Cu- and Co-TPtdBP molecules adsorbed in double-protrusion islands on Cu(111); in total, more than 50.000 molecules were categorized. The procedure was as follows: the STM images were printed on A4 paper, islands were marked, and the total number of the molecules determined. Then, the monobenzo molecules and monobenzo pairs in the bulk, and the fuzzy, monobenzo, and dibenzo edge terminations were marked with different colors and denoted next to the printed images; see Figure S4. Finally, the values from all 78 STM images were added together to form Table 1. The total time spent for manual counting was about 20 h."

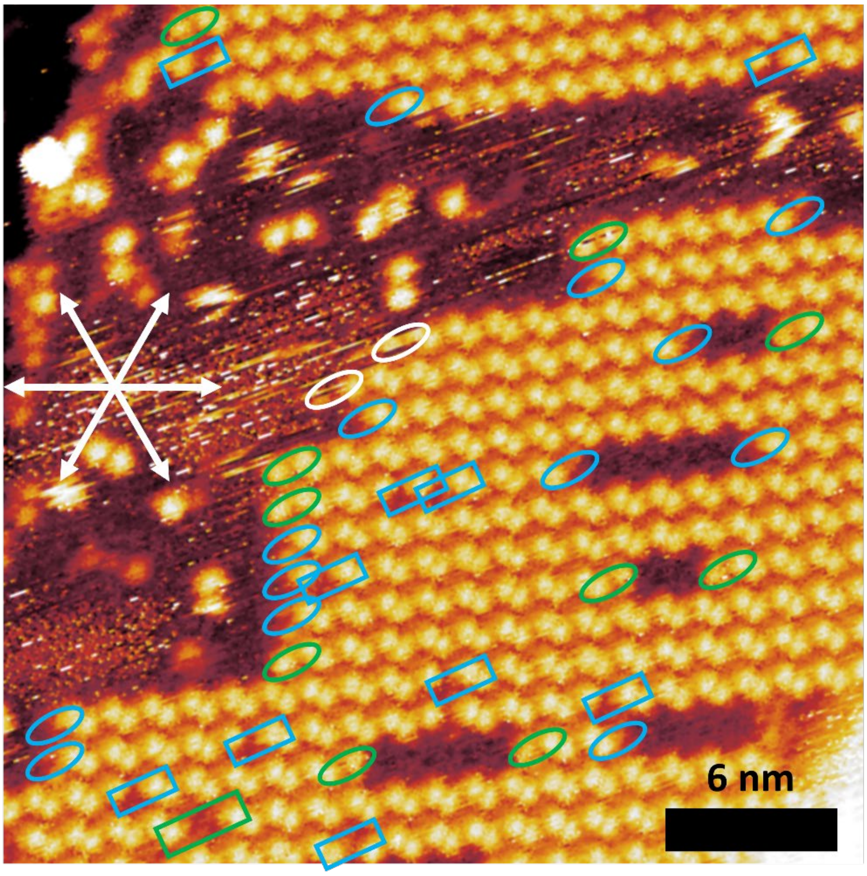

Total	Edges			Bulk	
	Monobenzo	Dibenzo	Fuzzy	Single Monobenzo	Monobenzo pair
244	12	10	2	10	1

Figure S4. Representative STM image of Cu-TPtdBP on Cu(111) with a total of 244 molecules in double-protrusion islands. The different marked features are: monobenzo edge terminations (12 blue ovals), dibenzo edge terminations (10 green ovals), fuzzy edges (2 white ovals), monobenzo in the bulk of the molecular rows (10 blue rectangles) and monobenzo pairs inside the bulk of the molecular rows (1 green rectangle). Two edge terminations (upper left corner) were not labeled because they could not be unequivocally assigned. The table below the image summarizes the numbers of the features recorded in this image.
