# Supplementary material for: Site Preferences of Copper and Cobalt Monobenzo Porphyrins in a Trans‐Dibenzo Adsorption Structure on Cu(111)
Source: Chemphyschem. 2025 Oct 28;26(22):e202500524. doi: 10.1002/cphc.202500524 (PMC12640668; doi:10.1002/cphc.202500524)
Supplement: Supplementary file 1 — Supplementary Material [file CPHC-26-e202500524-s001.zip › cphc70119-sup-0002-SuppData-S2.pptx]

## Slide 1
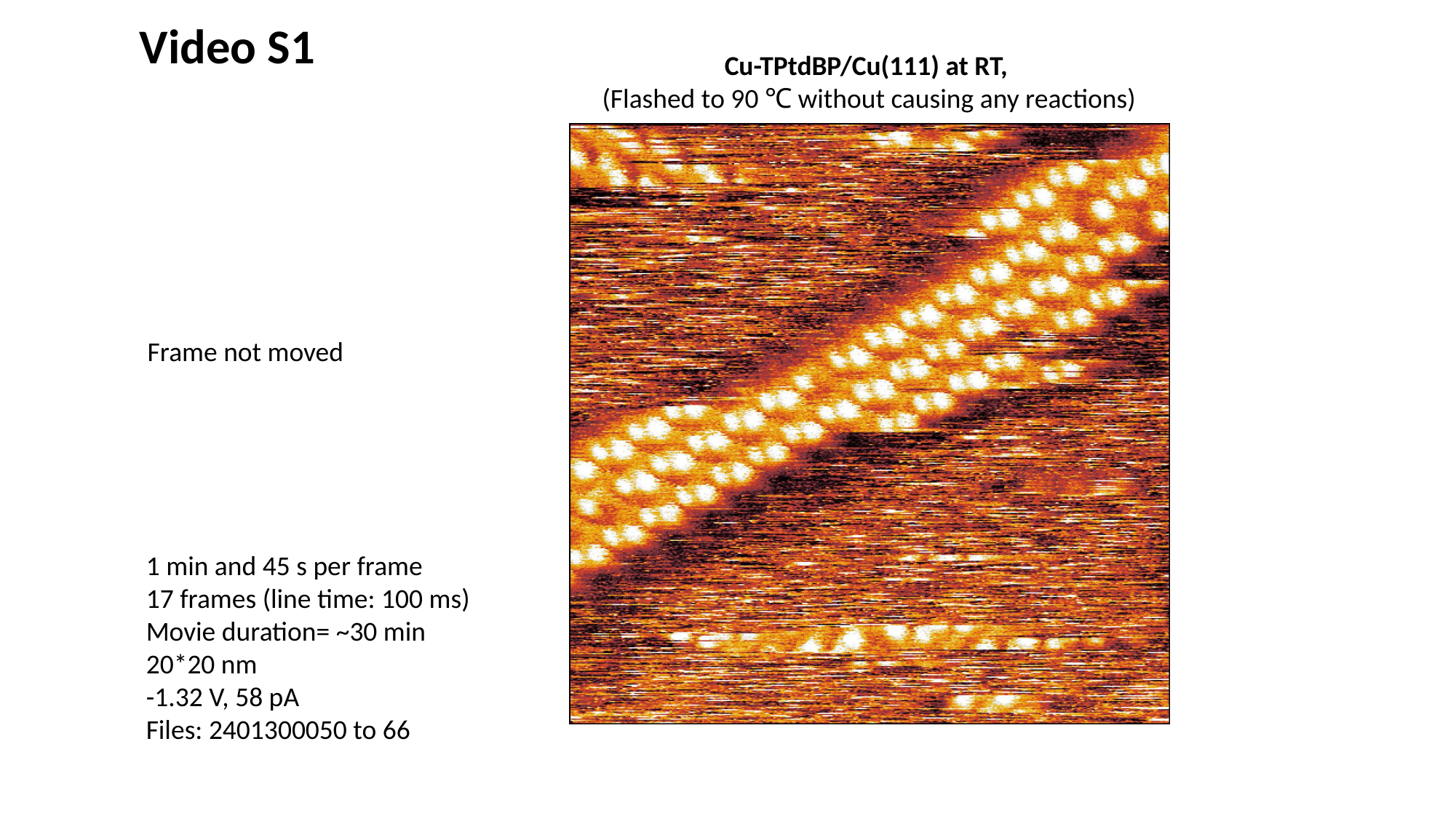

Video S1
Cu-TPtdBP/Cu(111) at RT,
(Flashed to 90 ℃ without causing any reactions)
Frame not moved
1 min and 45 s per frame
17 frames (line time: 100 ms)
Movie duration= ~30 min
20*20 nm
-1.32 V, 58 pA
Files: 2401300050 to 66

## Slide 2
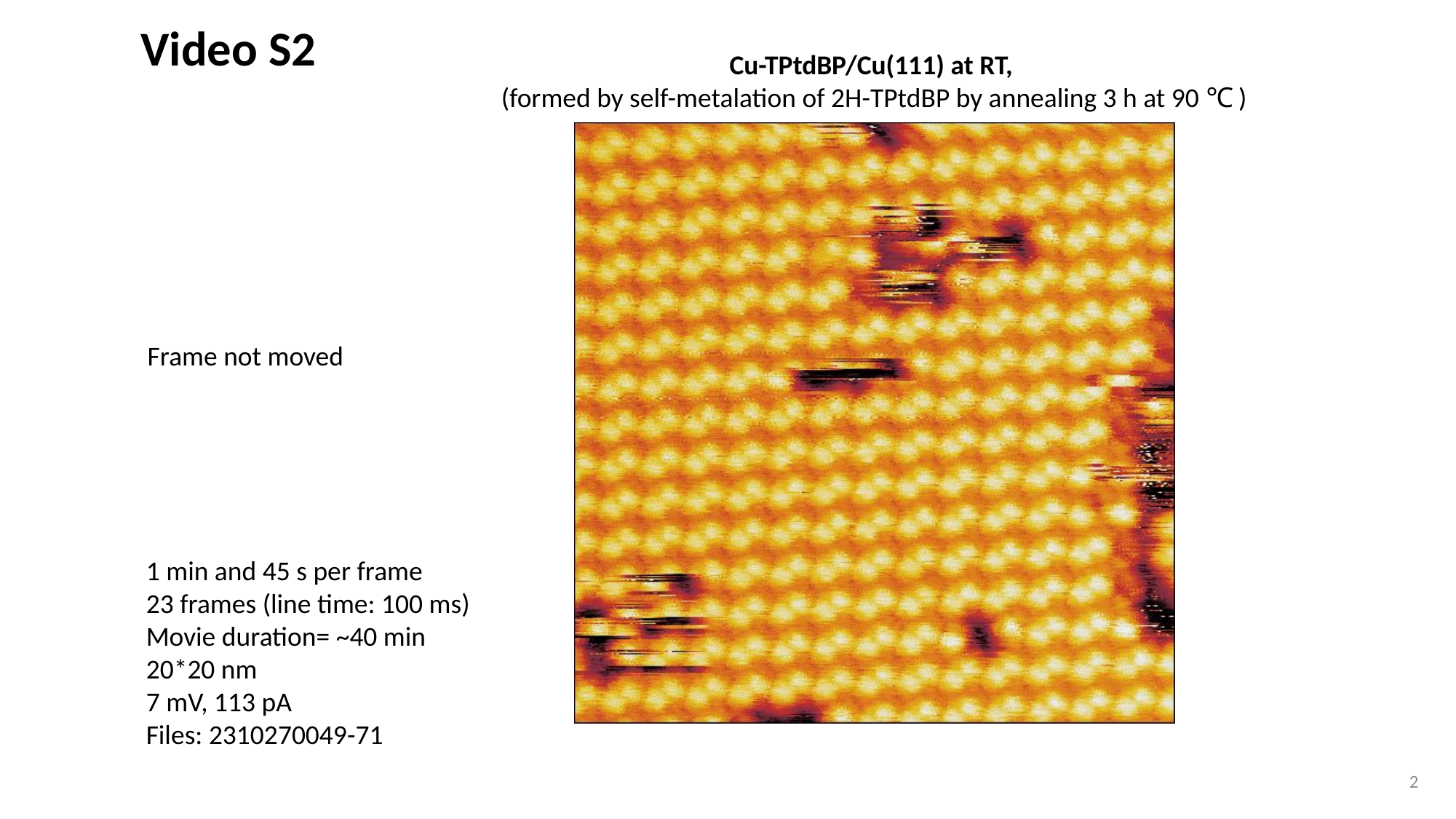

Video S2
Cu-TPtdBP/Cu(111) at RT,
(formed by self-metalation of 2H-TPtdBP by annealing 3 h at 90 ℃ )
Frame not moved
1 min and 45 s per frame
23 frames (line time: 100 ms)
Movie duration= ~40 min
20*20 nm
7 mV, 113 pA
Files: 2310270049-71
2
